# Low long-lasting insecticidal net use in malaria elimination areas in Southern Ethiopia: results from community based cross-sectional study

**DOI:** 10.1186/s12936-024-04909-y

**Published:** 2024-04-04

**Authors:** Misganu Endriyas, Mekidim Kassa, Yilma Chisha, Emebet Mekonnen, Tebeje Misganaw, Eskindir Loha, Ayalew Astatkie

**Affiliations:** 1 South Ethiopia Regional Health Bureau, Hawassa, Ethiopia; 2 College of Medicine and Health Sciences, Arbaminch University, Arbaminch, Ethiopia; 3 Sidama Regional Health Bureau, Hawassa, Ethiopia; 4 Centre for International Health, University of Bergen, Bergen, Norway; 5 Chr. Michelsen Institute, Bergen, Norway; 6 School of Public Health, College of Medicine and Health Sciences, Hawassa University, Hawassa, Ethiopia

**Keywords:** Malaria, Long-lasting insecticide-treated nets, Ownership, Utilization

## Abstract

**Background:**

Despite remarkable progress in malaria burden reduction, malaria continues to be a major public health problem globally. Ethiopia has been distributing long-lasting insecticidal nets (LLINs) for free and nationwide distribution was completed in 2016. However, evidence suggests that the utilization of LLINs varies from setting to setting and from time to time due to different factors, and up-to-date evidence is required for LLIN related decision-making. Hence, this study was designed to assess LLIN utilization and its determinants in the Southern Nations, Nationalities, and People’s Region (SNNPR) of Ethiopia.

**Methods:**

A community-based cross-sectional study was conducted in Southern Ethiopia in 2019. Using multi-stage sampling, a total of 2466 households were included. The region was stratified based on the annual malaria index as high, moderate, low, and free strata. Cluster sampling was then applied to select households from high, moderate, and low strata. Data on LLIN ownership, utilization and different determinant factors were collected using household questionnaire. SurveyCTO was used to collect data and data was managed using Stata 15. Descriptive statistics and multilevel mixed-effects logistic regression were performed to identify the determinants of utilization of LLINs. Effect measures were reported using adjusted odds ratio (AOR) with 95% CI.

**Results:**

From a total of 2466 households, 48.7% of households had at least one LLIN. LLIN adequacy based on family size was 23% while it was15.7% based on universal access and 29.2% based on sleeping space. From 1202 households that possessed LLIN(s), 66.0% of households reported that they slept under LLIN the night preceding the survey. However, when the total population in all surveyed households were considered, only 22.9% of household members slept under LLIN the night preceding the survey. Malaria endemicity, educational status, wealth status, and knowledge about malaria were associated with LLINs utilization. In addition, reasons for non-use included perceived absence of malaria, side effects of LLIN, conditions of LLINs, inconvenient space and low awareness.

**Conclusion:**

Low LLIN coverage and low utilization were noted. A low level of utilization was associated with malaria endemicity, wealth status and level of awareness. Distribution of LLIN and continuous follow-up with community awareness creation activities are vital to improve coverage and utilization of LLINs, and to ensure the country’s malaria elimination goal.

**Supplementary Information:**

The online version contains supplementary material available at 10.1186/s12936-024-04909-y.

## Background

Despite huge investments and efforts to respond to malaria, the number of malaria cases in 84 malaria endemic countries increased from 245 million in 2020 to 249 million in 2022 [1]. The highest burden of malaria occurs in the World Health Organization (WHO) African Region that accounted for about 95% of cases in 2020 [2].

In Ethiopia, more than 60% of total population are at risk of malaria [3]. In 2022, Ethiopia accounted for 2.1% of cases and 1.7% of deaths attributable to malaria globally [1]. In addition, malaria has been associated with loss of earnings, low school attendance and high treatment cost [3]. Based on annual parasite incidence per 1000 population and altitude, malarious areas have been stratified in to free (zero cases), low (less than five cases per 1000 population), moderate (between five and 100 cases per 1000 population) and high (more than 100 cases per 1000 population) [4]. However, the stratification is modified in 2020 as free (zero), very low (zero to five), low (five to ten), moderate (10–50) and high (50 plus) [5]. The national malaria elimination programme targeted low stratum as a starting point [4]. Malaria is endemic in the study setting and in 2017, it was the sixth top cause of morbidity [6]. The region implements malaria elimination programme in selected zones that have low annual malaria parasite incidence. In malaria elimination areas, activities are intensified. For example, surveillance includes active case finding using reactive case detection approach (testing and treating individuals adjacent to index case) and foci investigation. However, indoor residual spray (IRS) is recommended in high burden settings.

Recognizing the need to hasten progress in reducing the burden of malaria, different organizations have developed strategies for malaria that emphasizes the need for universal access to interventions for malaria prevention (including LLINs), diagnosis and treatment; that all countries should accelerate efforts towards malaria elimination [7–9]. Ethiopia achieved a universal distribution of LLINs in 2016 [10], with aim of covering all population in malarious areas and to intensify the intervention for malaria elimination. In 2017, additional replacement LLINs were distributed in the region.

Mapping malaria situations (like prevalence, incidence, intervention coverage, mortality, risk factors, etc.) is key for malaria programme decision-making [11–16]. Evidence suggests that LLIN utilization varies from setting to setting and from time to time due to different factors that comprises socioeconomic status like educational level, environmental factor like mosquito abundance and LLIN related factors like shape and color [17–19]. Studies on ownership, utilization and determinants of utilization of LLINs in Southern Ethiopia [20, 21] indicated varying level of LLIN utilization (77.0% in Wolaita zone versus 85.1% in Gamo zone). This indicates that up-to-date evidence is needed for effective monitoring and evaluation, and for decision-making [22]. Hence, this study was designed to assess LLIN utilization and its determinants, and thereby to contribute to evidence-based decision-making in SNNPR.

## Methods

### Study setting

A community based cross-sectional study was conducted from April to June 2019, which is the second largest (minor) malaria transmission season in Ethiopia [23]. The study was conducted in SNNPR, which was the third largest administrative region of Ethiopia representing about 20% of the country's population at the time of the study. It was the most diverse region in the country in terms of language, culture and ethnic background. Administratively, the region was divided into 17 zones, 1 city administration and 3 special woredas and had 76 hospitals of all type (9 private), 720 health centres (24 private) and 3878 health posts [24]. Woreda or district in the study setting is an administrative structure with approximate population of 100,000 while kebele is smallest administrative structure within woreda or town with approximate population of 5000. Currently, the region is divided in to four regions: Sidama, Southwest Ethiopia, South Ethiopia and Central Ethiopia regions.

### Sample size

Minimum sample size was calculated using single population proportion formula for cross-sectional study by considering the following assumptions: 68.3% of LLINs utilization from previous study conducted in South West Ethiopia [25], 5% level of significance and 4% margin of error. This yielded sample size of 520 households. Considering design effect of three for multi-stage sampling and adding 10% non-response rate, the minimum sample size estimated was 1716. However, 2466 households were included when all households in final clusters were considered.

### Sampling

**Fig. 1:**
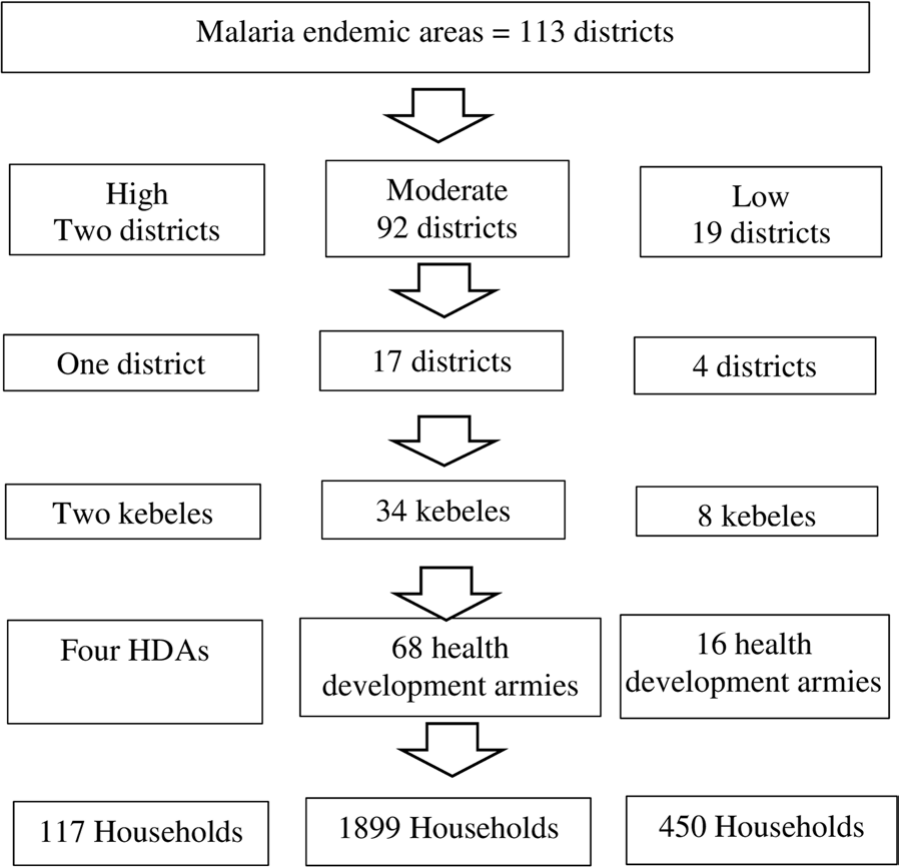
Schematic representation of sampling procedure

Multi-stage sampling technique was used to select study participants (Fig. 1). First, the region was stratified to high, medium, low and malaria free districts based on malaria endemicity. Districts with high, medium and low malaria endemicity were considered for sampling. Estimated sample size was distributed to each stratum proportionally considering total population in strata and random samples of districts were selected. From a total of 113 districts, one high, 17 moderate and four low strata, a total of 22 districts were selected. And at field level, after getting the lists of malarious kebeles in selected districts, two kebeles (a total of 44 kebeles) were selected from each district by lottery method. Similarly, after getting the lists of health development armies (HDAs) in respective kebeles, two HDAs were randomly selected from each kebele (a total of 88 HDAs or clusters). HDA is a development-oriented network of approximately 30 households in which one leader leads the rest of the households. The leader monitors the implementation of health packages by members of households. For example, LLIN utilization, antenatal care, and immunization. Finally, all households residing in selected HDAs for at least six months were interviewed. Respondents were household heads and in cases when heads were absent, elders of age above 18 years were included. The arrow in Fig. 1, except the last one that indicates cluster sampling, shows simple random sampling at each stage.

### Data collection tools and procedures

The semi-structured questionnaire for the study was adapted from similar study [21] and pre-tested. The questionnaire was prepared in English and translated to Amharic (regional working language) and then back to English by another expert to check consistency. Data were collected by 14 BSc nurses and six health officers while five MPH/MSc holders led the team as supervisors. The overall process was supervised by investigators. Training on the purpose of the study, how to interview and ethics was given for three days by the investigators.

Interviewers administered the questionnaire face to face in the selected households. Local guides led teams and translated in the case of language barriers. To minimize social desirability bias, respondents were told that the study was not designed to evaluate them, but the information was required to guide malaria programme. Data was collected by using SurveyCTO [26] on android based smart phones adjusted with security and skip patterns and data was synchronized daily to web-system.

### Variables

The dependent variable was LLIN utilization. The independent variables were categorized into individual or household and community level variables. Data on socio-demographic and economic characteristics of household, knowledge related to malaria and its prevention, sources of information for household about malaria and LLINs utilization, household maternal and child health services use in past 12 months (antenatal care (ANC), postnatal care (PNC), delivery, immunization, and growth monitoring), availability of bed net, sources of bed net and LLINs utilization were considered as individual/household variables. Residence, malaria endemicity (based on annual parasite incidence) and inclusion of the district for malaria elimination (by government malaria programme) were considered as community level variables.

LLIN ownership was assessed by interview and observation while LLINs utilization was assessed by report of sleeping of any family member under LLINs at night preceding the survey [27]. LLIN adequacy was determined by using different of perspectives: universal LLIN coverage recommended by the WHO (one LLINs for two people) [28] and national strategic plan [29]. The national strategic plan aims to achieve 100% coverage of one LLIN per sleeping space in malarious endemic area. A sleeping space is any space where family members sleep during night. The national routine LLINs distribution guideline recommends provision of LLINs based on family size indicating one LLIN for 1–2 people, 2 for 3–5 people, 3 for 6–7 people and 4 for 8 or more people.

### Data management

The excel file downloaded from SurveyCTO server was imported to Stata 15 [30] for statistical analysis. Data (Additional file 1) was transformed, and descriptive and regression analyses were performed. Wealth index was constructed separately for urban and rural settings using principal component analysis. For rural area, eight assets: land, cow, calf, ox, donkey, sheep, goat and chicken were used, while for urban setting nine items: TV, refrigerator, electricity, bed, wall, floor, phone, motor bicycle and roof were used. Finally, both urban and rural wealth categories were created separately and categorized into five groups ranging from poorest to richest. Knowledge about malaria and its prevention was assessed by using ten items (Table 2) and scoring mean or above mean was categorized as good knowledge while below mean was poor. Text responses were reviewed, coded, and categorized to similar responses.

Adequacy per sleeping space was determined by comparing the number of available LLIN with number of sleeping spaces in the households. If the number of LLIN was equal to or greater than number of sleeping spaces, it was recorded as “adequate”. To determine adequacy per family size, the number of LLIN required as per guideline was estimated by categorizing family size and this estimate was compared with the available LLIN. For universal LLIN coverage, number of household members was divided in to two (decimals were rounded to higher count numbers) and the results were compared with the number of LLIN available in the household. For LLIN utilization, the denominator was household with at least one LLIN. If any family member used LLIN the preceding night, the household was recorded “yes” to utilization at household level and the percentage of LLIN utilization indicates proportion of households where any individual was reported to use an LLIN (Additional file 2).

Multilevel mixed-effects logistic regression was used to identify the determinants of LLIN utilization. The multilevel model was used because multi-stage sampling was used and to account the intracluster correlation coefficient (ICC) at district level, which was 12%. The regression was performed considering households with at least one LLIN and done at household level. Independent variables with P-value of less than 0.25 in bivariable multilevel mixed-effects logistic regression were considered for multivariable logistic regression [31]. Associations between LLINs utilization and determinants were declared at p-value less than 5% and the effect of determinants was reported using adjusted odds ratio with 95% CI.

### Ethical considerations

Ethical approval for the study was obtained from the SNNPR Health Bureau ethical review committee (Ref £’6–19/31111). Letter of permission from the regional health bureau was dispatched to all selected districts and respective kebeles. Informed verbal consent was approved (as it is common in study setting), no personal identifier was collected and taken from all respondents and data was handled anonymously.

## Results

### Socio-demographic characteristics

A total of 2466 households from 22 districts were included with a response rate of 99.8%. One thousand two hundred and twelve (49.2%) households had more than five members, 858 (34.8%) of households were led by heads of age 30 or less (mean age was 37.9 ± 13.2); 1166 (47.4%) heads were not able to read and write, and 1326 (53.8%) heads were farmers (Table 1).

**Table 1:** Socio-demographic characteristics of study participants, SNNPR, 2019

Variable	Category	Frequency	Percent
Gender of household head	Male	1363	55.3
	Female	1103	44.7
Age of household head	≤ 30	858	34.8
	31–40	818	33.2
	≥ 41	790	32.0
Marital status of household head	Married	2209	89.6
	Widowed	198	8.0
	Divorced	31	1.3
	Single	28	1.1
Family size	Less than five	827	33.5
	Five or more	1639	66.5
Under five children present	No	1018	41.3
	Yes	1448	58.7
Educational status of household head	Can't read/write	1166	47.3
	Read and write	115	4.7
	Primary (1–8)	773	31.3
	Secondary (9–12)	272	11.0
	Certificate and above	140	5.7
Occupation of household head	Employed	136	5.5
	Student	44	1.8
	Farmer	1326	53.8
	Pastoralist	185	7.5
	Merchant	257	10.4
	Housewife	418	17.0
	Others	100	4.1
Wealth index	Poorest	493	20.0
	Second	493	20.0
	Middle	496	20.1
	Forth	490	19.9
	Richest	494	20.0
Overall house condition	Needs repair	1033	41.9
	Sound structure	1433	58.1
Malaria endemicity	High	117	4.7
	Moderate	1899	77.0
	Low	450	18.2
Total		2466	100.0

### Knowledge about malaria and its prevention

Ten items regarding malaria and its prevention were used to compute knowledge about malaria and its prevention. About three-fourths (74.5%) of respondents reported that malaria is public health problem, 94.8% reported the frequent mosquito biting time (night) and 94.8% said that there is modern medication to treat malaria. But, only 18.0% of participants clearly answered the most at-risk population (pregnant and children) and malaria peak seasons (Table 2). The mean score of knowledge of participants out of ten was 56.5% ± 17.5 and 1413 (57.3%) scored more than the mean and operationally called good knowledge.

**Table 2:** Knowledge about malaria and its prevention, SNNPR, 2019

Items	Frequency	Percent
Know malaria as public problem	1838	74.5
Know malaria transmission by mosquito bite	1667	67.6
Know malaria peak seasons	481	19.5
Know frequent mosquito biting time	2338	94.8
Know most at-risk population (pregnant and children)	444	18.0
Know signs and symptoms (headache, chills and fever)	937	38.0
Know malaria confirmation method (lab diagnosis)	1063	43.1
Know medications^a^	2337	94.8
Know at least one of major prevention methods (LLINs, IRS or environmental management)	1813	73.5
Know LLINs uses (kill and protect from insects)	1168	47.4

^a^Only about the use of modern medication, not specific anti-malaria medications were asked

### LLIN ownership and adequacy

From total households surveyed, 1202 (48.7%) had at least one LLIN (including old) that can serve. The distribution of ownership of LLINs among strata of malaria based on annual malaria parasite incidence (high, moderate and low) is presented in Tables 3. The distribution showed that more than three-fourths (76.9%) of the districts in high burden areas had at least one LLIN, from which 28.2% had three or more LLINs. Considering the adequacy of LLIN as per international and national targets, the proportion of households that had LLINs per universal LLINs coverage was 15.7%, per family size was 23.0% and per sleeping space was 29.2%.

**Table 3:** Number of LLINs owned by households across strata of districts based on malaria burden, SNNPR, 2019

Number of LLINs owned by households	Annual Parasite Incidence Status			Total No (%)
	High No (%)	Moderate No (%)	Low No (%)	
No LLINs	27 (23.1)	990 (52.1)	247 (54.9)	1264 (51.3)
One LLINs	21 (17.9)	350 (18.4)	85 (18.9)	456 (18.5)
Two LLINs	36 (30.8)	339 (17.9)	78 (17.3)	453 (18.3)
Three and more LLINs	33 (28.2)	220 (11.6)	40 (8.9)	293 (11.9)
Total	117 (100)	1899 (100)	450 (100)	2466 (100)

### LLINs utilization

From 1202 households that possessed LLIN(s), in 793 (66.0%) of the households, at least one person reportedly slept under LLINs the night preceding the survey, and only 22.9% of family numbers were protected (slept under LLINs). The proportion of households utilizing LLIN was lower in areas selected for malaria elimination (304 (62.8%)) as compared to non-malaria elimination areas (489 (68.1%)), which was also significant in regression analysis (Table 4).

**Table 4:** Multilevel logistic regression analysis to identify determinants of LLINs utilization, SNNPR, 2019

Variables	Categories	Model II COR 95% CI	Model III COR 95% CI	Model IV AOR 95% CI
Age category of household head	≤ 30	1		1
	31–40	1.00 [0.72–1.41]		1.02 [0.72–1.43]
	≥ 41	0.96 [0.66–1.40]		0.98 [0.67–1.43]
Used maternal or child health services	No	1		1
	Yes	1.00 [0.70–1.43]		1.00 [0.70–1.43]
Under five children present	No	1		1
	Yes	1.16 [0.84–1.60]		1.13 [0.82–1.57]
Educational status of household heads	Can’t read/write	1		1
	Read and write	1.50 [0.72–3.12]		1.57 [0.75–3.28]
	Primary (1–8)	1.70 [1.21–2.39]		1.80 [1.28–2.53]*
	Secondary (9–12)	1.47 [0.94–2.30]		1.54 [0.98–2.42]
	Above secondary level education	2.39 [1.08–5.32]		2.51 [1.13–5.60]*
Occupation of household heads	Employed	1		1
	Student	0.70 [0.20–2.44]		0.73 [0.21–2.53]
	Farmer	0.73 [0.32–1.67]		0.79 [0.34–1.79]
	Pastoralist	1.04 [0.38–2.85]		1.03 [0.38–2.79]
	Merchant	0.52 [0.21–1.24]		0.54 [0.22–1.29]
	Housewife	0.64 [0.28–1.50]		0.67 [0.29–1.56]
	Others	0.54 [0.21–1.37]		0.54 [0.22–1.39]
Wealth status	Poorest	1		1
	Second	0.81 [0.51–1.29]		0.83 [0.52–1.32]
	Middle	0.55 [0.36–0.86]		0.56 [0.36–0.87]*
	Forth	0.55 [0.35–0.89]		0.56 [0.35–0.89]*
	Richest	0.37 [0.22–0.60]		0.35 [0.22–0.57]*
Overall house condition	Maintenance needed	1		1
	Sound structure	0.78 [0.58–1.04]		0.77 [0.58–1.03]
Overall knowledge	Poor	1		1
	Good	1.76 [1.32–2.35]		1.69 [1.27–2.26]*
Malaria endemicity (strata)	High		1	1
	Moderate		0.24 [0.06–0.94]	0.21 [0.05–0.79]*
	Low		0.28 [0.07–1.79]	0.25 [0.06–1.02]
Selected for elimination	No		1	1
	Yes		0.52 [0.28–0.95]	0.48 [0.26–0.89]*

* statistically significant (*P* < 0.05)

### Reasons for not using

**Fig. 2:**
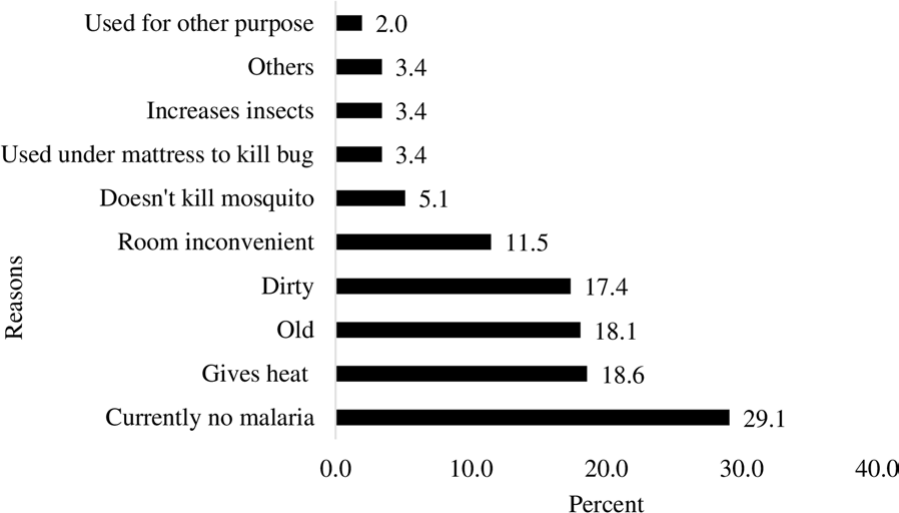
Reasons for not using LLINs, SNNPR, 2019

Those who were not using LLIN(s) (n = 409), were asked for the reasons why they were not using LLINs at the time of data collection and the reasons were summarized in Fig. 2. Three out of 10 were not using considering it was not malaria season and about one-fifth were not using believing that it is hot to sleep under LLINs (18.6%), the LLIN was old (18.1%), and the LLIN was dirty (17.4%).

### Determinants of LLINs utilization

Age, education and occupation of head of household, utilization of maternal or child health service in the past 12 months, existence of under five children in the family, wealth index, overall structure of house, overall knowledge about malaria and its prevention, malaria endemicity in the district, and consideration of the district for malaria elimination were considered for multivariable multilevel mixed-effects logistic regression model. However, finally, education of head of household, wealth index, overall knowledge about malaria and its prevention, malaria endemicity in the district, and consideration of the district for malaria elimination were found to be determinants of LLIN utilization.

Households in moderate malaria burden setting had 79% (AOR: 0.21; 95% CI 0.05–0.79) lesser odds to utilize LLIN than households in the high malaria burden area. Households in districts included in national malaria elimination programme had 52% (AOR: 0.48; 95% CI 0.26–0.89) lesser odds of utilizing LLIN as compared to households in districts not included in national malaria elimination programme.

Households with heads who had completed primary education were 80% (AOR: 1.80; 95% CI 1.28–2.53) more likely to utilize LLINs than those who cannot read and write. Similarly, households with heads who had completed above secondary level education were 2.51 times (AOR: 2.51; 95% CI 1.13–5.60) more likely to utilize LLINs than those who cannot read and write. Moreover, households who had good knowledge about malaria and its prevention were 69% (AOR: 1.69; 95% CI 1.27–2.26) more likely to use LLIN than those who had poor knowledge.

Households belonging to middle wealth category were 44% (AOR: 0.56; 95% CI 0.36–0.87) less likely to use LLIN than the poorest. Likewise, households belonging to the fourth category of wealth category were 44% (AOR: 0.56; 95% CI 0.35–0.89) and richest were 65% (AOR: 0.35; 95% CI 0.22–0.57) less likely to use LLINs than the poorest.

### Goodness-of-fit of the multilevel model

The Intraclass Correlation Coefficient (ICC) in the null model was (0.13), which means that 13% of the variability of LLIN utilization was due to the differences between clusters or unobserved factors at the community level. This indicates that the multilevel logistic regression model is best to estimate the predictors of LLIN utilization among households in the region than single-level logistic regression model. The Akaike’s Information Criteria (AIC) is the smallest in model 4 (AIC = 1462.09) as compared to other models. Thus, model 4 is the best-fitting model. Therefore, all interpretations and reports were made based on this model. In addition, the median odds ratio (MOR) in all models was greater than one noting that there is variation in the LLIN utilization among households between community levels in the region. The proportional change in variance (PCV) value in the fourth model showed that about 36.17% of the variability of LLIN utilization was explained or determined by both the individual-level and community-level predictors (Table 5).

**Table 5:** Model summary of multilevel logistic regression analysis

Parameter	Null (model I)	Model II	Model III	Model IV
ICC	0.13	0.13	0.09	0.08
Variance (SE)	0.47 (0.19)	0.48 (0.21)	0.33 (0.15)	0.30 (0.15)
PCV	Reference	− 0.021	0.298	0.362
MOR	1.92	1.93	1.73	1.68
AIC	1497.18	1463.30	1497.12	1462.09
BIC	1507.36	1575.32	1522.57	1589.39
DIC (− 2Log-likelihood)	48.29	37.93	34.18	21.89

## Discussion

The study showed that about half of households (48.7%) had at least one LLINs, near to two thirds (66.0%) of households owning LLINs were using the LLINs and only less than one-fourth (22.9%) of household members were utilizing it. The utilization of LLIN was associated with educational status, wealth index, malaria endemicity, and knowledge about malaria. Moreover, LLIN utilization was low in malaria elimination setting was lower than non-malaria elimination programme area.

The ownership of LLINs in terms of universal coverage (15.7%), national target based on family size (23.0%) and per sleeping space (29.2%) were found low as compared to the WHO recommendation that dictates at least 80% of universal LLIN coverage. The result is also much lower than the national plan to cover 100% of sleeping spaces in malarious area [28, 29]. The utilization of LLINs at household level was found to be 66% and only less than one-fourth (22.9%) of household members were using it. As Ethiopia is implementing malaria elimination, this gap of access to LLIN should be taken in to account and prioritized as malaria elimination requires intensified response than malaria burden reduction phase [32]. The survey was conducted two years later after mass campaign for LLIN distribution. The finding may indicate shorter durability of LLINs, and the government needs close monitoring to improve LLIN life span.

The level of LLIN utilization varies from context to context and influenced by different factors. The systematic review conducted on LLIN use in Sub-Saharan Africa summarized these factors as the education level of the head of the household, wealth quintile, the number of under five children in the household, and the knowledge that sleeping under a mosquito net protects against malaria [33]. Another systematic review on factors influencing LLIN utilization in sub-Saharan Africa specifically among under five children reported that cost, inadequate number, hotness of the weather, absence of visible mosquitoes, rooms designs, unaffordability, insufficient knowledge on causes of malaria, poor attitude to use, color of LLIN, chemicals use, odour and shape of LLIN are influencing LLIN utilization [34]. In current study, educational status, wealth status, malaria endemicity, and knowledge about malaria were found to be associated LLINs utilization.

Households in moderate malaria burden areas were less likely to utilize LLINs as compared to households in high burden area. Similarly, households in districts included in national malaria elimination programme as compared to households in districts not included in national malaria elimination programme were less likely to utilize LLINs. This could be due to low perceived risk of malaria and lesser amount of mosquitoes in low malaria settings [17]. As Ethiopia aims malaria elimination, this should be taken into account to improve consistent LLIN utilization for the success of the programme because the alternative vector control, IRS, is not recommended in low malaria stratum settings in Ethiopia [4, 5]. To achieve malaria elimination, the WHO recommends that the national malaria programmes need to ensure that all population at risk of malaria are protected through the provision of, use and timely replacement of LLINs [35].

Households with heads who had completed primary and above secondary level education were more likely to utilize LLINs than those who cannot read and write. In addition, those who had good knowledge about malaria and its prevention were more likely to use LLINs than those who had poor knowledge. This finding was in line with other studies that reported higher educational level and good knowledge are positive factors for LLIN use [33, 36, 37].

Households belonging to middle, fourth and richest category were less likely to use LLINs than households belonging to poorest category. The association between wealth status and LLIN utilization has shown both positive and negative relationships [38]. As a study done in Ghana [38] previously reported, households in the richest quintile were less likely to have all members sleep under LLINs. This could be due to the reason that rich families may consider other prevention methods and this might have affected the LLIN utilization. For example, wealthier people who live in houses with door and window screens may think that they are protected from mosquito bites and may not use LLIN even if they have them in their households [39].

Although the study included different strata of malarious area and large number of clusters in samples to make results more generalizable to the settings, due to the nature of cross-sectional study, the level of LLIN ownership and utilization reported in this study indicates only the situation at the time of data collection and may not represent other times as levels highly vary over time. In addition, social desirability bias could have affected the responses, and some households could falsely report that they had slept under LLINs the day preceding the survey. Furthermore, although total population that slept in the house the preceding night was collected during data collection, the study was limited in capturing profile of LLIN users and running regression at individual level.

## Conclusion

Both the ownership and utilization of LLIN were low in the study area. Malaria endemicity, educational status, wealth status, and knowledge about malaria were associated with LLINs utilization. More emphasis is required in malaria elimination settings for the success of the programme. To ensure universal LLIN coverage and optimal utilization, more LLIN distribution and follow-up are recommended.

### Supplementary Information

**Additional file 1: Data S1.** Data in Stata dta file type.

**Additional file 2: **Utilization of long-lasting insecticide nets and associated factors in SNNPR.

## Data Availability

All relevant data are within the manuscript and supplementary file.
