# Supplementary material for: Low long-lasting insecticidal net use in malaria elimination areas in Southern Ethiopia: results from community based cross-sectional study
Source: Malar J. 2024 Apr 4;23:94. doi: 10.1186/s12936-024-04909-y (PMC10996104; doi:10.1186/s12936-024-04909-y)
Supplement: Supplementary file 2 — Additional file 2: Utilization of long-lasting insecticide nets and associated factors in SNNPR. [file 12936_2024_4909_MOESM2_ESM.pdf]

Title: Utilization of long-lasting insecticide nets and associated factors in SNNPR

**Household information panel**	
H1. Zone: ___ ___ ___	H2. District/town: ___ ___
H3. CLUSTER TYPE:<br>Urban... 1<br>Agrarian.. 2<br>Pastoralist 3	H4. Kebele: ________________________<br>H5. Date of interview: ___ /___ /_______
H6. Interviewer name and sign:<br>Name __________________________ ________	H7. Supervisor name and sign:<br>Name___________________________ _________

CONSENT AND CONFIDENTIALITY

GOOD DAY! MY NAME IS ______ AND MY FRIEND'S NAME IS ______. WE ARE FROM SNNPR HEALTH BUREAU AND ARBAMINCH UNIVERSITY. WE ARE WORKING ON A PROJECT CONCERNED WITH OWNERSHIP AND UTILIZATION OF LONG-LASTING INSECTICIDE NETS AND ASSOCIATED FACTORS AMONG HOUSEHOLDS IN SNNPR. I WOULD LIKE TO TALK TO YOU ABOUT THESE SUBJECTS. THE INTERVIEW WILL TAKE ABOUT (***number***) MINUTES. ALL THE INFORMATION WE OBTAIN WILL REMAIN STRICTLY CONFIDENTIAL AND YOUR ANSWERS WILL NEVER BE SHARED WITH ANYONE OTHER THAN OUR PROJECT TEAM.

MAY I START NOW?

- ☐ *Yes, permission is given* ⇨ *go to question 1.*
- ☐ *No, permission is not given* ⇨END INTERVIEW. *Discuss this result with your supervisor.*

**Household characteristics**			
1	What is the sex of head of this household?	*Male* ....1<br>*Female* 2	
2	What is the age of head of this household?	____________________________________<br>Don't know 99	
3	What is the marital status of head of this household?	*Married* ....*1*<br>*Widowed*..*2*<br>*Divorced*..*3*<br>*Single*..*4*	
4	Total number of household members	*Total number of household members* ...._______<br>*Total number of under five children* ...._______<br>*Total number of pregnant women* .._______	
5	Total number of household members who slept in the house in previous night	*Total number of household members* .... _______<br>*Total number of under five children* ...._______<br>*Total number of pregnant women* .._______	
6	What is the religion of the head of this household?	*Orthodox* ....*1*<br>*Protestant*..*2*<br>*Catholic*...*3*<br>*Islam* ....*4*<br>*Others (specify)* ....____________________	

		No religion *99*	
7	What is educational status of head of family?	*Can't read and write* ....1<br>*Read and write* ....2<br>*Primary* ....3<br>*Secondary* ....4<br>*Certificate and above* ....5<br>Don't know ________________________99	
8	What is occupation of head of this household?	*Office worker*..1<br>*Student*...2<br>*Farmer*...3<br>*Pastoralist* ....4<br>*Merchant* ....5<br>*House wife*..6<br>*Other (specify)*..............________________	
9	What is average monthly income of the family?	________________________________<br>Don't know 99	
10	What is the health care facility that is nearest to you?	Health post ....1<br>Health center ....2<br>Public/private hospital ....3<br>Private clinic ....4<br>Other (specify) ....__________________<br>Don't know 99	
11	How long does it take you from your home to reach the nearest public health care facility in minute?	Distance in minutes ....______	
12	What transport do you use commonly to go there?	Walk on foot ....1<br>Bus ....2<br>Bajaj ....3<br>Motorbike ....4<br>Use animal transport ....5<br>Others (specify) ....______	
13	Do you have exposure to the following maternal and child health care services UTILIZATION? (Circle all available)	ANC ....1<br>Family planning ....2<br>Delivery ....3<br>Postnatal care ....4<br>Immunization ....5<br>Others (specify) ....__________	
14	how many sleeping places (beds, mats, etc.) does your household have (indoors and outdoors)?	Total ...._______<br>Indoor ...._______<br>Outdoor ...._______	
15	At any time in the last 6 months, has anyone come into your house to spray indoor residuals against mosquito?	Yes ....1<br>No...2<br>Don't know 99	2,99 →19
16	If yes, how long ago was it sprayed?<br>(if less than 1 month, fill 1 month)	Month ....___	

17	At any time in the last 6 months, have the walls of your house been plastered or painted?	Yes ....1<br>No....2<br>Don't know 99	2,99 →19
18	How long ago was it plastered or painted?<br>(if less than 1 month, fill 1 month)	Month ....___	
KNOWLEDGE AND PERCEPTIONS ABOUT MALARIA			
19	Do you think malaria is a major health problem in this community?	Yes ....1<br>No....2<br>Don't know 99	
20	Can malaria be transmitted from one person to another?	Yes ....1<br>No....2<br>Don't know 99	
21	How can a person acquire malaria?<br>(Don't read the list, circle all responses that apply)	By breathing ....1<br>By mosquito bite ....2<br>By body contact with malaria patient....3<br>By drinking dirty water ....4<br>Being exposed to bad/cold air ....5<br>Exposure to dirty swampy areas ....6<br>By getting socked in rain ....7<br>By eating immature maize/sugar cane ....8<br>By hunger ....9<br>By lack of personal hygiene ....10<br>By evil spirit ....11<br>Other (specify) ________<br>Don't know 99	
22	In a given year, at what season/s is the probability of getting malaria is higher?<br>(Don't read the list, circle all responses that apply)	Winter ....1<br>Spring ....2<br>Summer ....3<br>Fall ....4<br>Equal in all seasons ....5<br>I don't know 99	
23	When do mosquitoes usually bite a person?	Day ....1<br>Evening ....2<br>Night ....3<br>Day and night ....4<br>Don't know 99	
24	To which group of the population malaria is more serious?	Adults ....1<br>Children ....2<br>Pregnant women ....3<br>Elderly ....4<br>Pregnant women/children ....5<br>Equally serious for all ....6<br>Don't know 99	
25	What are the main signs and symptoms of malaria? (Don't read the list, circle all responses that apply)	Fever ....1<br>Shivering/chills ....2<br>Sweating ....3<br>Headache 4	

		Vomiting ....5<br>Diarrhea ....6<br>Loss of appetite ....7<br>Bitterness in the mouth ....8<br>Weakness/tiredness ....9<br>Splenomegaly ....10<br>Backache ....11<br>Anemia ....12<br>Convulsion ....13<br>Thirsty ....14<br>Joint pain ....15<br>Other (specify) ________________________<br>Don't know 99	
26	How is it possible to know certainly that someone is infected by malaria?<br>(Don't read the list, circle all applicable)	By symptoms only ....1<br>By laboratory diagnosis ....2<br>By traditional healers ....3<br>Is not possible ....4<br>Others(specify) _______________________<br>I don't know 99	
27	Is malaria a curable disease with treatment?	Yes ....1<br>No...2<br>Don't know 99	
28	How malaria can be cured?<br>(Don't read the list, circle all applicable)	Home-made remedies ....1<br>Modern medicines ....2<br>Traditional healers ....3<br>Other (Specify) ....___________________<br>I don't know 99	2,99 →29
29	Commonly, where do you go first to treat illness with a fever or malaria?	Prepare remedy/remedies homemade ....1<br>Go to health institutions ....2<br>Go to drug venders ....3<br>Others(specify) _______________________<br>I don't know 99	
30	Do you think that malaria is preventable?	Yes ....1<br>No...2<br>Don't know 99	2,99 →33
31	What are the different malaria preventive measures that you know? (Don't read list, circle all responses that apply)	Eating good food ....1<br>Keeping house clean ....2<br>Remaining indoors at night ....3<br>Sleeping under a mosquito net ....4<br>Spraying house with insecticide ....5<br>Spraying house with aerosols ("flit") ....6<br>Smoking in the house (fumigation) ....7<br>Applying ointment/repellents on the skin ..8<br>Draining mosquito breeding sites ....9<br>Window screening ....10<br>Other (specify)___________________<br>Don't know 99	

32	What are the different malaria preventive measures that you practice? (Don't read list, circle all responses that apply)	Eating good food ....1<br>Keeping house clean ....2<br>Remaining indoors at night ....3<br>Sleeping under a mosquito net ....4<br>Spraying house with insecticide ....5<br>Spraying house with aerosols ("flit") ....6<br>Smoking in the house (fumigation) ....7<br>Applying ointment/repellents on the skin ..8<br>Draining mosquito breeding sites ....9<br>Window screening ....10<br>Other (specify)___________________<br>Don't know 99	
33	Can sleeping under "mosquito net" protect a person from malaria?	Yes ....1<br>No...2<br>Don't know 99	
34	In your opinion, what is the average duration of "mosquito net" service years?	Year ....____	
35	Did anyone tell you about the uses of ITNs?	Yes ....1<br>No...2	2 →37
36	From where you heard the uses of ITNs? (Don't read list, circle all responses that apply)	Health worker...1<br>Radio ....2<br>TV ....3<br>Internet ....4<br>School..5<br>Friends...6<br>Family member ....7<br>Newspaper (pamphlet) ....8<br>Community meeting (HDA) ....9<br>Other (specify) ...._________________<br>Don't know 99	
37	What do you are uses of ITNs? (Don't read list, circle all responses that apply)	Protect from insects' bite ....1<br>Kill insects ....2<br>Other (specify) ...._________________<br>Don't know 99	
38	What are other uses you think of ITNs than protecting from insects? (Don't read list, circle all responses that apply)	To mangle "Kocho" ....1<br>To make rope (tie) ....2<br>As kerchief (scarf) ....3<br>For packing materials ....4<br>Others (specify) ....__________________<br>Don't know 99	
ITN POSSESSION AND UTILIZATION			
39	Does your household currently possess any mosquito nets to sleep under?	Yes ....1<br>No...2	2 →53
40	How many mosquito nets do you currently possess?	Old with holes ...._____<br>Used tight ...._____<br>New not used ...._____	
41	What is the shape of your LLINs? (observe)	Rectangular ...._____	

		Conical ...._____	
42	What is the colour of your LLINs? (observe)	White ...._____<br>Green ...._____<br>Blue ...._____	
43	Where did you obtain nets?<br>(Don't read list, circle all responses that apply)	Provided by health facility ....1<br>Provided by Woreda Health Office ....2<br>Provided by NGO ....3<br>Bought from market/shop ....4<br>From friends ....5<br>Other (specify) ___________<br>Don't know 99	
44	How many of the nets the household possess are hanged over the bed/mat during the interview?	______________	
45	How many of the nets the household have are currently used by household members while sleeping?	________________	
46	How long ago did your household obtain the most recent mosquito net?	Year ....___<br>Month ....___	
47	Did you (your family member) sleep under a mosquito net during the previous night?	Yes ....1<br>No..2	1 →49
48	What are reasons that household member didn't sleep under LLINs on the previous night preceding the survey?<br>(Don't read the list, circle all responses that apply)	*Sleeping under LLINs is not convenient .....1*<br>*I sold it ....2*<br>*I used it for other purposes ....3*<br>*No malaria during this time of the year .....4*<br>*Doesn't kill mosquitos ....5*<br>*Used under mattress to kill bug ....6*<br>*It gives too warm to sleep under it.............7*<br>*Inconvenient to easily get up during night..8*<br>*Tucking the net every night is boring..........9*<br>*It gives you skin irritation.........................10*<br>*Others (specify)................. _____________*	ANY OPTION →50
49	How many of the people who slept in this household in the previous night slept under a net, including you?	*Total number of household members. _____*<br>*Total number of under five children ..._____*<br>*Total number of pregnant women ......_____*	
50	If you have only one "mosquito net", to whom would you give priority using it?<br>(Circle only one answer)	*Husband ....1*<br>*Wife ....2*<br>*Husband and wife ....3*<br>*Wife with youngest child ....4*<br>*Young children ....5*<br>*Elderly/grandparents ....6*<br>*Pregnant women ....7*<br>*Other (specify)________________*<br>*Don't know 99*	
51	Have you ever experienced any problems when using "mosquito nets"?	Yes ....1<br>No..2	2 →55

52	If Yes, what problems have you or your family members experienced?<br>(Don't read the list, circle all responses that apply)	*It gives too warm to sleep under it* ....*1*<br>*Mosquitoes still bite you through it* ....*2*<br>*Inconvenient to easily get up during night* .*3*<br>*Tucking the net every night is boring* ....*4*<br>*It gives you skin irritation* ....*5*<br>*Other (specify)*..............________________	ANY OPTION→ 55
53	If your household possess currently no mosquito net, have your household ever had it before?	Yes ....1<br>No...2<br>Don't know 99	2, 99 →55
54	If your household possesses no mosquito net, what are the reasons for not having it?<br>(Don't read the list, circle all responses that apply)	Not convenient while sleeping ....1<br>Not aware of its use ....2<br>Not know where to get it ....3<br>It is unavailable ....4<br>It has a side effect since treated ....5<br>Does not prevent malaria ....6<br>Has become old or lost ....7<br>Not adequate space to hang it in the house 8<br>Doesn't kill mosquito ....9<br>Other (specify) _________________<br>Don't know 99	
55	What is the shape of ITN you desire to have?	Rectangular ....1<br>Conical 2	
56	What is the colour of ITN you desire to have?	White ....1<br>Green ....2<br>Blue 3	
**Malaria morbidity**			
57	Has anyone in your family experienced signs and symptoms of malaria in past 3 months?	Yes ....1<br>No...2<br>Don't know 99	2,99 →61
58	If yes, how many people experienced this illness?	Number of people ....____	
59	How did you confirm that signs and symptoms were of malaria?	Self-suspect ....1<br>Confirmed by provider 2	
60	Form where did sick person seek care?<br>(Don't read the list, circle all responses that apply)	Home ....1<br>Health post ....2<br>Health center ....3<br>Hospital ....4<br>Private clinic ....5<br>Drug vendor ....6<br>Traditional healer ....7<br>Other (specify) _________________<br>Don't know 99	
**Household structure and assets**			
61	Does your household have:<br>(Put only number of functional materials)	Radio ....____<br>Bicycle ....____<br>Motor cycle ....____<br>Car/truck ....____	

		Kerosene lamp ....____<br>Television ....____<br>Phone including mobile ....____<br>Cart ....____<br>Grain-mill ....____<br>Refrigerator ....____<br>Sewing machine ....____<br>Electricity supply ....____<br>Solar ....____<br>Bed ....____	
62	Does your household own the following? (Quantify each of the items in appropriate measurement)	Farming land (hectare) ....____<br>Cow ....____<br>Calf ....____<br>Ox ....____<br>Horse ....____<br>Mule ....____<br>Donkey ....____<br>Sheep ....____<br>Goat ....____<br>Chicken ....___	
63	The walls are made mainly of:	Wood only ....1<br>Wood and mud ....2<br>Mud bricks ....3<br>Cement blocks ....4<br>Sticks ....5<br>Thatch ....6<br>Other (specify) _________________	
64	The floor is made mainly of:	Earth/mud ....1<br>Cement ....2<br>Cement tiles ....3<br>Other (specify)_________________	
65	The roof is made mainly of:	Thatch/grass ....1<br>Corrugated iron sheets ....2<br>Plastic ....3<br>Other (specify)_________________	
66	Does the house have windows?	Yes ....1<br>No..2	
67	If "yes", do they are they screened and/or covered by curtains or others?	Yes ....1<br>No..2	
68	What is the observed structural condition of the main dwelling?	Seriously dilapidated ....1<br>Needs major repair ....2<br>Sound structure 3	

We thank you for your time and response!!
